# Use of Respiratory Syncytial Virus Vaccines in Older Adults: Recommendations of the Advisory Committee on Immunization Practices — United States, 2023

**DOI:** 10.15585/mmwr.mm7229a4

**Published:** 2023-07-21

**Authors:** Michael Melgar, Amadea Britton, Lauren E. Roper, H. Keipp Talbot, Sarah S. Long, Camille N. Kotton, Fiona P. Havers

**Affiliations:** ^1^Coronavirus and Other Respiratory Viruses Division, National Center for Immunization and Respiratory Diseases, CDC; ^2^Vanderbilt University School of Medicine, Nashville, Tennessee; ^3^Drexel University College of Medicine, Philadelphia, Pennsylvania; ^4^Harvard Medical School, Boston, Massachusetts.

**Summary**

**What is already known about this topic?**

Respiratory syncytial virus (RSV) causes substantial morbidity and mortality in older adults. In May 2023, the Food and Drug Administration approved the first two vaccines for prevention of RSV lower respiratory tract disease (LRTD) for use in adults aged ≥60 years.

**What is added by this report?**

For both vaccine products, vaccination with a single RSV vaccine dose demonstrated moderate to high efficacy in preventing symptomatic RSV-associated LRTD among adults aged ≥60 years. On June 21, 2023, the Advisory Committee on Immunization Practices recommended that persons aged ≥60 years may receive a single dose of RSV vaccine, using shared clinical decision-making.

**What are the implications for public health practice?**

RSV vaccination might prevent substantial morbidity in older adults at risk for severe RSV disease; postmarketing surveillance for safety and effectiveness will direct future guidance.

## Abstract

Respiratory syncytial virus (RSV) is a cause of severe respiratory illness in older adults. In May 2023, the Food and Drug Administration approved the first vaccines for prevention of RSV-associated lower respiratory tract disease in adults aged ≥60 years. Since May 2022, the Advisory Committee on Immunization Practices (ACIP) Respiratory Syncytial Virus Vaccines Adult Work Group met at least monthly to review available evidence regarding the safety, immunogenicity, and efficacy of these vaccines among adults aged ≥60 years. On June 21, 2023, ACIP voted to recommend that adults aged ≥60 years may receive a single dose of an RSV vaccine, using shared clinical decision-making. This report summarizes the body of evidence considered for this recommendation and provides clinical guidance for the use of RSV vaccines in adults aged ≥60 years. RSV vaccines have demonstrated moderate to high efficacy in preventing RSV-associated lower respiratory tract disease and have the potential to prevent substantial morbidity and mortality among older adults; postmarketing surveillance will direct future guidance.

## Introduction

In the United States, respiratory syncytial virus (RSV) causes seasonal epidemics of respiratory illness. Although the COVID-19 pandemic interrupted seasonal RSV circulation, the timing and number of incident cases of the 2022–23 fall and winter epidemic suggested a likely gradual return to prepandemic seasonality (*1*).

Each season, RSV causes substantial morbidity and mortality in older adults, including lower respiratory tract disease (LRTD), hospitalization, and death. Incidence estimates vary widely and are affected by undertesting and potentially low sensitivity of standard diagnostic testing among adults (*2*–*5*). Most adult RSV disease cases occur among older adults with an estimated 60,000–160,000 hospitalizations and 6,000–10,000 deaths annually among adults aged ≥65 years (*5*–*10*).

Adults with certain medical conditions, including chronic obstructive pulmonary disease, asthma, congestive heart failure, coronary artery disease, cerebrovascular disease, diabetes mellitus, and chronic kidney disease, are at increased risk for RSV-associated hospitalization (*11*–*13*), as are residents of long-term care facilities (*14*), and persons who are frail* or of advanced age (incidence of RSV-associated hospitalization among adults increases with age, with the highest rates among those aged ≥75 years) (*6*,*15*). RSV can also cause severe disease in persons with compromised immunity, including recipients of hematopoietic stem cell transplantation and patients taking immunosuppressive medications (e.g., for solid organ transplantation, cancer treatment, or other conditions) (*16*,*17*).

In May 2023, the Food and Drug Administration (FDA) approved the first vaccines for prevention of RSV-associated LRTD in adults aged ≥60 years. RSVPreF3 (Arexvy, GSK) is a 1-dose (0.5 mL) adjuvanted (AS01_E_) recombinant stabilized prefusion F protein (preF) vaccine (*18*). RSVpreF (Abrysvo, Pfizer) is a 1-dose (0.5 mL) recombinant stabilized preF vaccine (*19*).

## Methods

Since May 2022, CDC’s Advisory Committee on Immunization Practices (ACIP) RSV Vaccines Adult Work Group (Work Group) met at least monthly to review available evidence regarding the safety, immunogenicity, and efficacy of the GSK and Pfizer RSV vaccines among adults aged ≥60 years. A systematic review of published and unpublished evidence of the efficacy and safety of these vaccines among persons aged ≥60 years was conducted. The body of evidence consisted of one phase 3 randomized controlled trial and one combined phase 1 and 2 (phase 1/2) randomized controlled trial for each vaccine. The Work Group used the Grading of Recommendations, Assessment, Development and Evaluation (GRADE) approach to independently determine the certainty of evidence for outcomes related to each vaccine, rated on a scale of high to very low certainty.^†^ In evaluating safety, the Work Group defined inflammatory neurologic events as cases of Guillain-Barré syndrome (GBS), chronic inflammatory demyelinating polyneuropathy, and acute central nervous system inflammation (e.g., transverse myelitis or acute disseminated encephalomyelitis [ADEM]) occurring within 42 days after vaccination. The Work Group then employed the Evidence to Recommendation Framework to guide its deliberations on recommendation for RSV vaccination, reviewing data on the public health problem, benefits and harms, value to the target population, acceptability to key stakeholders, feasibility, resource use, and equity.^§^ Work Group conclusions regarding evidence for the use of RSV vaccines among adults aged ≥60 years were presented to ACIP at public meetings on February 23 and June 21, 2023 (*10*,*15*).

## Vaccine Efficacy and Safety

###  GSK Vaccine

Evaluated efficacy evidence for the GSK RSV vaccine consisted of data from one ongoing randomized, double-blind, placebo-controlled phase 3 clinical trial conducted in 17 countries and including 24,973 immunocompetent participants aged ≥60 years randomized 1:1 to receive 1 dose of vaccine (intervention group, 120 *μ*g preF protein with AS01_E_ adjuvant) or saline placebo (control group) (*20*). Efficacy findings were based on analyses of data collected during May 2021–March 2023, which included two complete RSV seasons for Northern Hemisphere participants and one complete RSV season for Southern Hemisphere participants. Efficacy analyses for season one spanned May 2021–April 2022, while efficacy analyses for season two spanned August 2022–March 2023; exact study-defined season dates were site-dependent. Mean time from vaccination to end of efficacy follow-up across both seasons was approximately 15 months per participant.

The efficacy of 1 dose of the GSK vaccine in preventing symptomatic, laboratory-confirmed RSV-associated LRTD^¶^ was 82.6% (96.95% CI = 57.9%–94.1%) during the first RSV season and 56.1% (95% CI = 28.2%–74.4%) during the second season (Table 1).** Efficacy of 1 dose over two seasons was 74.5% (97.5% CI = 60.0%–84.5%) in preventing RSV-associated LRTD and 77.5% (95% CI = 57.9%–89.0%) in preventing medically attended RSV-associated LRTD.^††^ The study was not powered to estimate efficacy against hospitalization (intervention group = one event; control group = five events), severe RSV illness requiring respiratory support (intervention group = one event; control group = five events),^§§^ or death (no events).^¶¶^

**TABLE 1:** Efficacy of 1 dose of GSK respiratory syncytial virus RSVpreF3 vaccine against respiratory syncytial virus–associated disease among adults aged ≥60 years — multiple countries, 2021–2023

Efficacy evaluation period	Vaccine efficacy against outcome*	
	RSV-associated LRTD^†^	RSV-associated medically attended LRTD^§^
Season 1^¶^	82.6 (57.9–94.1)**	87.5 (58.9–97.6)^††^
Season 2^§§^	56.1 (28.2–74.4)^††^	—^¶¶^
Combined seasons 1 and 2 (interim)***	74.5 (60.0–84.5)^†††^	77.5 (57.9–89.0)^††^

**Abbreviations:** LRTD = lower respiratory tract disease; RSV = respiratory syncytial virus.

* Manufacturer-calculated efficacy. Includes events >14 days after injection and person-time available from the manufacturer’s pivotal phase 3 trial. Estimates adjusted for participant age and region.

^†^ LRTD defined as two or more lower respiratory symptoms (new or increased sputum, cough, and dyspnea) or signs (new or increased wheezing, crackles or rhonchi detected during chest auscultation, respiratory rate ≥20 respirations per minute, low or decreased oxygen saturation [<95% or ≤90% if baseline was <95%], and need for oxygen supplementation) for ≥24 hours, including one or more lower respiratory signs, or three or more lower respiratory symptoms for ≥24 hours.

^§^ Medically attended RSV-associated LRTD defined as LRTD plus attention at one or more inpatient or outpatient health care services. Estimates were not included in per-protocol assessments.

^¶^ Season 1 vaccine efficacy estimates reflect efficacy against first events occurring during the first complete RSV season for Northern Hemisphere participants and a partial first RSV season for Southern Hemisphere participants (May 2021–April 2022; exact study-defined season dates were site-dependent).

** 96.95% CI; the CI for primary trial endpoint was adjusted for multiplicity.

^††^ 95% CI.

^§§^ Season 2 vaccine efficacy estimates reflect efficacy against first events occurring during the second complete Northern Hemisphere RSV season for Northern Hemisphere participants (August 2022–March 2023; exact study-defined season dates were site-dependent). In addition to Northern Hemisphere participants, Southern Hemisphere participants were also included in these analyses, but this time span reflects an interseason period with low RSV incidence in the Southern Hemisphere.

^¶¶^ Interim analysis underpowered to estimate efficacy.

*** Combined season 1 and 2 (interim) vaccine efficacy estimates reflect efficacy against first events occurring any time during Season 1 or Season 2. The mean time from start to end of efficacy surveillance was approximately 15 months per participant.

^†††^ 97.5% CI; the CI for primary trial endpoint was adjusted for multiplicity.

Evidence regarding safety of the GSK vaccine consisted of data from two randomized, double-blind, placebo-controlled clinical trials, including the same ongoing phase 3 trial (*20*) and a phase 1/2 trial with 201 participants aged ≥60 years who received either the vaccine formulation used in phase 3 or placebo (*21*). Across both clinical trials, severe reactogenicity events (grade 3 solicited local or systemic reactions recorded during days 0–4 [phase 3 trial] and days 0–7 [phase 1/2 trial] after vaccination) occurred in 3.8% of the intervention group participants, compared with 0.9% of the control group participants (pooled relative risk [RR] = 4.10; 95% CI = 1.99–8.45) (Table 2). The frequency of serious adverse events (SAEs)*** across both trials was similar in the intervention (4.4%) and control (4.3%) groups (pooled RR = 1.02; 95% CI = 0.91–1.15). A higher number of participants in the intervention group than in the control group reported atrial fibrillation as an unsolicited event within the 30 days after injection (intervention = 10 events [0.1%]; control = four events [<0.1%]), eight of which were SAEs [intervention = seven; control = one]; three of the SAEs corresponded to new onset atrial fibrillation (intervention = two; control = one) (*22*).

**TABLE 2:** Safety* of 1 dose of GSK respiratory syncytial virus RSVPreF3 vaccine in adults aged ≥60 years — multiple countries, 2021–2023

Safety event	Risk for event		
	RSVPreF3 recipients no./No. (%)^†^	Placebo recipients no./No. (%)^§^	Relative risk (95% CI)^¶^
Serious AE**	549/12,570 (4.4)	540/12,604 (4.3)	1.02 (0.91–1.15)
Severe reactogenicity events^††^	37/979 (3.8)	9/976 (0.9)	4.10 (1.99–8.45)
Inflammatory neurologic events^§§^	3 events in trials without placebo recipients^¶¶^	—^¶¶^	—^¶¶^

**Abbreviations:** AE = adverse event; GBS = Guillain-Barré syndrome.

* Includes serious adverse events and severe reactogenicity events observed in GSK’s pivotal phase 3 trial (https://pubmed.ncbi.nlm.nih.gov/36791160/) and phase 1/2 trial (https://pubmed.ncbi.nlm.nih.gov/35904987/). Inflammatory neurologic events include those observed across all GSK clinical trials, including an open-label study (https://clinicaltrials.gov/ct2/show/NCT04732871) and a coadministration study (https://clinicaltrials.gov/ct2/show/NCT04841577). Additional data provided by GSK.

^†^ Represents number of events and percentage of all participants experiencing events observed among RSVPreF3 vaccine recipients across all included trials for each outcome.

^§^ Represents number of events and percentage of all participants experiencing events observed among placebo recipients across all included trials for each outcome.

^¶^ Pooled relative risk for events in all included trials for each outcome.

** Serious AEs were defined as any untoward medical occurrence (during 6 months after injection in the phase 3 trial and 60 days after injection in the phase 1/2 trial) that resulted in death, was life threatening, required inpatient hospitalization or prolongation of existing hospitalization, resulted in persistent disability or incapacity, or was a congenital anomaly or birth defect.

^††^ Severe reactogenicity events were defined as grade 3–solicited local reaction (injection site pain, redness and swelling) or systemic reactions (fatigue, fever, headache, gastrointestinal symptoms [nausea, vomiting, diarrhea, or abdominal pain], arthralgia, myalgia, and shivering) recorded during days 0–4 after vaccination in the phase 3 trial and days 0–7 after vaccination in the phase 1/2 trial. For injection site redness and swelling, grade 3 corresponded to a diameter >3.9” (>100 mm). For fever, grade 3 corresponded to a temperature >102.2°F (>39°C). For all other reactions, grade 3 corresponded to reactions that prevented normal, everyday activities. Grade 4 events were not defined in these trials.

^§§^ Defined by the Advisory Committee on Immunization Practices Respiratory Syncytial Virus Vaccines Adult Work Group as GBS (including GBS variants), chronic inflammatory demyelinating polyneuropathy, or acute central nervous system inflammation (e.g., transverse myelitis or acute disseminated encephalomyelitis) occurring ≤42 days after vaccination.

^¶¶^ No inflammatory neurologic events were reported in either the phase 3 or phase 1/2 trials. However, across all RSVPreF3 trials inflammatory neurologic events were reported in three of 17,922 adults vaccinated with RSVPreF3. Events included one case of GBS in an open-label phase 3 clinical trial, and two cases of acute disseminated encephalomyelitis among participants in a randomized phase 3 study of coadministration of RSVPreF3 and standard dose seasonal influenza vaccine. Relative risk could not be calculated because neither trial had a placebo-controlled comparator group.

Across all GSK vaccine clinical trials in older adults, inflammatory neurologic events were reported in three of 17,922 participants within 42 days after receipt of the GSK vaccine (*23*). All three events occurred in trials excluded from GRADE because of lack of an unvaccinated comparator arm. The reported cases included one case of GBS in a participant aged 78 years from Japan with symptom onset 9 days postvaccination in an open-label phase 3 clinical trial and two cases of ADEM among participants in a randomized phase 3 coadministration study (*15*,*22*). The two ADEM cases were reported in participants aged 71 years from the same site in South Africa after concomitant receipt of the GSK vaccine and standard dose seasonal influenza vaccine; symptom onset occurred 7 and 22 days postvaccination, and one case was fatal. In both ADEM cases, the diagnosis was based on symptoms and clinical findings only; diagnostic testing (including brain imaging, cerebrospinal fluid testing, and nerve conduction studies) was not performed, leading to uncertainty in the diagnoses. The investigator in the fatal case later revised the diagnosis from ADEM to hypoglycemia and dementia (*15*,*22*).

### Pfizer Vaccine

Evaluated efficacy evidence for the Pfizer vaccine consisted of data from one ongoing, randomized, double-blind, placebo-controlled phase 3 clinical trial conducted in seven countries and including 36,862 immunocompetent participants aged ≥60 years randomized 1:1 to receive 1 dose of vaccine (intervention group, 120 *μ*g preF protein) or placebo containing the same buffer ingredients as the vaccine but without active components (control group) (*24*). Efficacy findings were based on analyses of data collected during August 2021–January 2023, which included one complete RSV season for Northern and Southern Hemisphere participants and a partial second season for Northern Hemisphere participants only. Efficacy analyses for season one spanned August 2021–October 2022, while efficacy analyses for season two spanned July 2022–January 2023; exact study-defined season dates were site-dependent. Mean follow-up time from vaccination to end of efficacy follow-up across both seasons, including a gap in RSV surveillance between the first and second RSV seasons, was approximately 12 months per participant.

Efficacy of 1 dose of the Pfizer vaccine in preventing symptomatic, laboratory-confirmed RSV-associated LRTD^†††^ was 88.9% (95% CI = 53.6%–98.7%) during the first RSV season and 78.6% (95% CI = 23.2%–96.1%) during the partial second season (Table 3).^§§§^ Efficacy of a single dose over two seasons was 84.4% (95% CI = 59.6%–95.2%) in preventing RSV-associated LRTD and 81.0% (95% CI = 43.5%–95.2%) in preventing medically attended RSV-associated LRTD.^¶¶¶^ The study was not powered to estimate efficacy against hospitalization (intervention group = one event; control group = three events), severe RSV illness requiring respiratory support (intervention group = one event; control group = one event),**** or death (no events).^††††^

**TABLE 3:** Efficacy of 1 dose of Pfizer respiratory syncytial virus RSVpreF vaccine against respiratory syncytial virus–associated disease among adults aged ≥60 years — multiple countries, 2021–2023

Efficacy evaluation period	Vaccine efficacy against outcome, % (95% CI)*	
	RSV-associated LRTD^†^	RSV-associated medically attended LRTD^§^
Season 1^¶^	88.9 (53.6–98.7)	84.6 (32.0–98.3)
Season 2 (interim)**	78.6 (23.2–96.1)	—^††^
Combined seasons 1 and 2 (interim)^§§^	84.4 (59.6–95.2)	81.0 (43.5–95.2)

**Abbreviations:** LRTD = lower respiratory tract disease; LRTI = lower respiratory tract illness; RSV = respiratory syncytial virus.

* Manufacturer-calculated efficacy. Includes events >14 days after injection and person-time available from the manufacturer’s pivotal phase 3 trial. Estimates are unadjusted.

^†^ The RSVpreF trial had two co-primary endpoints, defined as RSV LRTI with two or more lower respiratory signs or symptoms lasting >1 day, and RSV LRTI with three or more lower respiratory signs or symptoms lasting >1 day. Lower respiratory signs and symptoms included new or worsened cough, sputum production, wheezing, shortness of breath, and tachypnea. For RSVpreF estimates in this report, LRTD refers to the RSVpreF trial endpoint of RSV LRTI with three or more lower respiratory signs or symptoms.

^§^ Medically attended RSV-associated LRTD was defined as LRTD prompting any health care visit (any outpatient or inpatient visit such as hospitalization, emergency department visit, urgent care visit, home health care services, primary care physician office visit, pulmonologist office visit, specialist office visit, other visit, or telehealth contact). Estimates were not included in per-protocol assessments.

^¶^ Season 1 vaccine efficacy estimates reflect efficacy against first events occurring during the first complete RSV season for Northern and Southern Hemisphere participants (August 2021–October 2022; exact study-defined season dates were site-dependent).

** Season 2 (interim) vaccine efficacy estimates reflect efficacy against first events occurring during the second complete RSV season for Northern Hemisphere participants only (July 2022–January 2023; Southern Hemisphere data not yet available).

^††^ Interim analysis underpowered to estimate efficacy.

^§§^ Combined season 1 and 2 (interim) vaccine efficacy estimates reflect efficacy against first events occurring any time during season 1 or season 2. The mean time from start to end of efficacy surveillance was approximately 12 months per participant.

Evidence regarding safety of the Pfizer vaccine consisted of data from two randomized, double-blind, placebo-controlled clinical trials, including the same ongoing phase 3 trial (*24*), and a phase 1/2 trial with 91 participants aged ≥65 years who received either the vaccine formulation used in phase 3 or placebo (*25*). Across both clinical trials, severe reactogenicity events (grade 3 or higher local or systemic reactions recorded during days 0–7 after vaccination) occurred in 1.0% of the intervention group participants, compared with 0.7% of the control group participants (pooled RR = 1.43; 95% CI = 0.85–2.39) (Table 4). The frequency of SAEs across both trials was similar in the intervention (4.3%) and control (4.1%) groups (pooled RR = 1.04; 95% CI = 0.94–1.15). A higher number of participants in the intervention group than in the control group reported atrial fibrillation as an unsolicited event within the 30 days after injection (intervention = 10 events [<0.1%]; control = four events [<0.1%], of which seven were SAEs [intervention = four; control = three]). Among participants who reported atrial fibrillation, a medical history of atrial fibrillation was reported by six of 10 Pfizer vaccine recipients and two of four placebo recipients (*26*).

**TABLE 4:** Safety* of 1 dose of Pfizer respiratory syncytial virus RSVpreF vaccine in adults aged ≥60 years — multiple countries, 2021–2023

Safety event	Risk for event		
	RSVpreF recipients no./No. (%)^†^	Placebo recipients no./No. (%)^§^	Relative risk (95% CI)^¶^
Serious AE**	792/18619 (4.3%)	749/18334 (4.1%)	1.04 (0.94–1.15)
Severe reactogenicity events^††^	36/3673 (1.0%)	24/3491 (0.7%)	1.43 (0.85–2.39)
Inflammatory neurologic events^§§^	3/18622 (—)^¶¶^	0/18335 (—)	—^¶¶^

**Abbreviations:** AE = adverse events; GBS = Guillain-Barré syndrome.

* Safety events observed in Pfizer’s pivotal phase 3 trial (https://pubmed.ncbi.nlm.nih.gov/37018468/) and phase 1/2 trial (https://pubmed.ncbi.nlm.nih.gov/34932102/). There were no additional inflammatory neurologic events observed in any Pfizer clinical trials other than the two trials included. Additional data provided by Pfizer.

^†^ Represents number of events and percent of all participants experiencing events observed among RSVpreF vaccine recipients across phase 3 and phase 1/2 trials.

^§^ Represents number of events and percent of all participants experiencing events observed among placebo recipients across phase 3 and phase 1/2 trials.

^¶^ Pooled relative risk for events in phase 3 and phase 1/2 trials.

** Serious AEs were defined as any untoward medical occurrence (during all available follow-up time [safety follow-up through February 2023] after injection in the phase 3 trial and 60 days for the phase 1/2 trial) that resulted in death, was life threatening, required inpatient hospitalization or prolongation of existing hospitalization, resulted in persistent disability or incapacity, or was a congenital anomaly or birth defect.

^††^ Severe reactogenicity events were defined as grade 3 or higher local reaction (injection site pain, redness and swelling) or systemic reaction (fever, fatigue or tiredness, headache, nausea, muscle pain, joint pain, vomiting, diarrhea, and other systemic event) recorded during days 0–7 after vaccination. For injection site redness and swelling, grade 3 corresponded to a diameter >3.9” (>100 mm) from e-diary or severe grade from adverse event case report form. For fever, grade 3 corresponded to a temperature >102°F (>38.9°C) from e-diary or severe grade from adverse event case report form. For all other reactions, grade 3 corresponded to reactions that prevented normal, everyday activities. Grade 4 event corresponded only to a fever >104°F (>40°C).

^§§^ Defined by the Advisory Committee on Immunization Practices Work Group as GBS (including GBS variants), chronic inflammatory demyelinating polyneuropathy, or acute central nervous system inflammation (e.g., transverse myelitis or acute disseminated encephalomyelitis) occurring ≤42 days after vaccination.

^¶¶^ Across all RSVpreF clinical trials, including trials other than the phase 3 and phase 1/2 trials summarized in this table, inflammatory neurologic events were reported in three of 20,255 adults ≤42 days after vaccination with RSVpreF (all in the phase 3 trial). The events included GBS, Miller Fisher syndrome (a GBS variant), and undifferentiated motor-sensory axonal polyneuropathy. Relative risk could not be calculated because no events were observed in the placebo-controlled comparator group.

Across all Pfizer vaccine clinical trials among older adults, inflammatory neurologic events were reported in three of 20,255 participants within 42 days after receipt of the vaccine (*15*,*26*,*27*). The events included GBS in a participant aged 66 years from the United States with symptom onset 14 days postvaccination; Miller Fisher syndrome (a GBS variant) in a participant aged 66 years from Japan with symptom onset 10 days postvaccination; and undifferentiated motor-sensory axonal polyneuropathy with worsening of preexisting symptoms 21 days postvaccination in a participant aged 68 years from Argentina (*15*,*26*,*27*).

## Rationale for Recommendations

Vaccination with a single dose of the GSK or Pfizer RSV vaccines demonstrated moderate to high efficacy in preventing symptomatic RSV-associated LRTD over two consecutive RSV seasons among adults aged ≥60 years. Although trials were underpowered to estimate efficacy against RSV-associated hospitalization and death, prevention of LRTD, including medically attended LRTD, suggests that vaccination might prevent considerable morbidity from RSV disease among adults aged ≥60 years.

Although both vaccines were generally well-tolerated with an acceptable safety profile, six cases of inflammatory neurologic events (including GBS, ADEM, and others) were reported after RSV vaccination in clinical trials. Whether these events occurred due to chance, or whether RSV vaccination increases the risk for inflammatory neurologic events is currently unknown. Until additional evidence becomes available from postmarketing surveillance clarifying the existence of any potential risk, RSV vaccination in older adults should be targeted to those who are at highest risk for severe RSV disease and therefore most likely to benefit from vaccination. The recommendation for shared clinical decision-making is intended to allow flexibility for providers and patients to consider individual risk for RSV disease, while taking into account patient preferences.

## Recommendations for Use of RSV Vaccines in Older Adults

On June 21, 2023, ACIP recommended that adults aged ≥60 years may receive a single dose of RSV vaccine, using shared clinical decision-making.^§§§§^

### Clinical Guidance

**Shared Clinical Decision-Making for Adults Aged ≥60 years. **Unlike routine and risk-based vaccine recommendations, recommendations based on shared clinical decision-making do not target all persons in a particular age group or an identifiable risk group. For RSV vaccination, the decision to vaccinate a patient should be based on a discussion between the health care provider and the patient, which might be guided by the patient’s risk for disease and their characteristics, values, and preferences; the provider’s clinical discretion; and the characteristics of the vaccine.

As part of this discussion, providers and patients should consider the patient’s risk for severe RSV-associated disease. Epidemiologic evidence indicates that persons aged ≥60 years who are at highest risk for severe RSV disease and who might be most likely to benefit from vaccination include those with chronic medical conditions such as lung diseases, including chronic obstructive pulmonary disease and asthma; cardiovascular diseases such as congestive heart failure and coronary artery disease; moderate or severe immune compromise (either attributable to a medical condition or receipt of immunosuppressive medications or treatment)^¶¶¶¶^; diabetes mellitus; neurologic or neuromuscular conditions; kidney disorders, liver disorders, and hematologic disorders; persons who are frail; persons of advanced age; and persons with other underlying conditions or factors that the provider determines might increase the risk for severe RSV-associated respiratory disease (Box). Adults aged ≥60 years who are residents of nursing homes and other long-term care facilities are also at risk for severe RSV disease. It should be noted that the numbers of persons enrolled in the trials who were frail, were of advanced age, and lived in long-term care facilities were limited, and persons with compromised immunity were excluded (some of whom might have an attenuated immune response to RSV vaccination). However, adults aged ≥60 years in these populations may receive vaccination using shared clinical decision-making given the potential for benefit.

BOX **Underlying medical conditions and other factors associated with increased risk for severe respiratory syncytial virus disease**

**Chronic underlying medical conditions associated with increased risk**

• Lung disease (such as chronic obstructive pulmonary disease and asthma)

• Cardiovascular diseases (such as congestive heart failure and coronary artery disease)

• Moderate or severe immune compromise*

• Diabetes mellitus

• Neurologic or neuromuscular conditions

• Kidney disorders

• Liver disorders

• Hematologic disorders

• Other underlying conditions that a health care provider determines might increase the risk for severe respiratory disease

**Other factors associated with increased risk**

• Frailty^†^

• Advanced age^§^

• Residence in a nursing home or other long-term care facility

• Other underlying factors that a health care provider determines might increase the risk for severe respiratory disease

**Abbreviation:** RSV = respiratory syncytial virus.

* A list of potentially immune compromising conditions is available at https://www.cdc.gov/coronavirus/2019-ncov/need-extra-precautions/people-who-are-immunocompromised.html.

^†^ Frailty is a multidimensional geriatric syndrome and reflects a state of increased vulnerability to adverse health outcomes. Although there is no consensus definition, one frequently used tool is the Fried frailty phenotype in which frailty is defined as a clinical syndrome with three or more of the following symptoms present: unintentional weight loss (10 lbs [4.5 kg] in the past year), self-reported exhaustion, weakness (grip strength), slow walking speed, and low physical activity.

^§^ Among adults aged ≥60 years, RSV incidence increases with advancing age. Although age may be considered in determining an older adult patient’s risk for severe RSV-associated disease, there is no specific age threshold at which RSV vaccination is more strongly recommended within the age group of adults aged ≥60 years.

### RSV Vaccination Timing

RSV vaccination is currently approved and recommended for administration as a single dose; sufficient evidence does not exist at this time to determine the need for revaccination. Optimally, vaccination should occur before the onset of the RSV season; however, typical RSV seasonality was disrupted by the COVID-19 pandemic and has not returned to prepandemic patterns. For the 2023–24 season, clinicians should offer RSV vaccination to adults aged ≥60 years using shared clinical decision-making as early as vaccine supply becomes available and should continue to offer vaccination to eligible adults who remain unvaccinated.

### Vaccine Administration, Including Coadministration with Other Vaccines

Coadministration of RSV vaccines with other adult vaccines during the same visit is acceptable.***** Available data on immunogenicity of coadministration of RSV vaccines and other vaccines are currently limited. Coadministration of RSV and seasonal influenza vaccines met noninferiority criteria for immunogenicity with the exception of the FluA/Darwin H3N2 strain when the GSK RSV vaccine was coadministered with adjuvanted quadrivalent inactivated influenza vaccine (*28*,*29*). RSV and influenza antibody titers were somewhat lower with coadministration; however, the clinical significance of this is unknown.

Administering RSV vaccine with one or more other vaccines at the same visit might increase local or systemic reactogenicity. Data are only available for coadministration of RSV and influenza vaccines, and evidence is mixed regarding increased reactogenicity. Data are lacking on the safety of coadministration with other vaccines that might be recommended for persons in this age group, such as COVID-19 vaccines; pneumococcal vaccines; adult tetanus, diphtheria, and pertussis vaccines; and the recombinant zoster vaccine (the recombinant zoster vaccine and GSK’s RSV vaccine contains the same adjuvant). When deciding whether to coadminister other vaccines with an RSV vaccine, providers should consider whether the patient is up to date with currently recommended vaccines, the feasibility of the patient returning for additional vaccine doses, risk for acquiring vaccine-preventable disease, vaccine reactogenicity profiles, and patient preferences. Postlicensure efficacy and safety monitoring of coadministered RSV vaccines with other vaccines will further direct guidance.

### Precautions and Contraindications

As with all vaccines, RSV vaccination should be delayed for persons experiencing moderate or severe acute illness with or without fever (precaution). RSV vaccines are contraindicated for and should not be administered to persons with a history of severe allergic reaction, such as anaphylaxis, to any component of the vaccine (*30*,*31*).

## Reporting of Vaccine Adverse Events

Adverse events after vaccination should be reported to the Vaccine Adverse Event Reporting System (VAERS). Reporting is encouraged for any clinically significant adverse event even if it is uncertain whether the vaccine caused the event. Information on how to submit a report to VAERS is available at https://vaers.hhs.gov/index.html or by telephone at 1-800-822-7967.

## Future Research and Monitoring Priorities

CDC will monitor adverse events, including cases of GBS, ADEM, and other inflammatory neurologic events after RSV vaccination through VAERS and the Vaccine Safety Datalink https://www.cdc.gov/vaccinesafety/ensuringsafety/monitoring/vsd/index.html). CDC will also prioritize estimating vaccine effectiveness against RSV-associated hospitalization. These data will be evaluated by CDC and ACIP as soon as they are available.

According to FDA postmarketing requirements and commitments, GSK will conduct a study evaluating risk for GBS, ADEM, and atrial fibrillation after vaccination with RSVPreF3 (*18*). Pfizer will conduct two studies, one evaluating risk for GBS and a second evaluating risk for atrial fibrillation after vaccination with RSVpreF (*19*). Pfizer will also evaluate the safety and immunogenicity of a second RSVpreF dose in a subset of participants in the main phase 3 trial; GSK will evaluate safety, immunogenicity, and efficacy of RSVPreF3 revaccination as part of its main phase 3 trial.

## Footnotes

* Frailty is a multidimensional geriatric syndrome and reflects a state of increased vulnerability to adverse health outcomes. Although there is no consensus definition, one frequently used tool is the Fried frailty phenotype in which frailty is defined as a clinical syndrome with three or more of the following signs or symptoms: unintentional weight loss (10 lbs [4.5 kg] in the past year), self-reported exhaustion, weakness (grip strength), slow walking speed, and low physical activity.

† GRADE tables are available online for both the GSK RSV vaccine (https://www.cdc.gov/vaccines/acip/recs/grade/GSK-Adjuvanted-RSVPreF3-adults.html) and the Pfizer RSV vaccine (https://www.cdc.gov/vaccines/acip/recs/grade/Pfizer-Bivalent-RSVpreF-adults.html). For the GSK RSV vaccine, the efficacy estimates presented differ slightly from efficacy estimates included in the GRADE tables because the manufacturer used a different method from CDC to calculate vaccine efficacy. Estimates in this report are those of the manufacturer, and estimates in the GRADE tables are those calculated by CDC.

§ Evidence to Recommendation documents are available for the GSK vaccine (https://www.cdc.gov/vaccines/acip/recs/grade/GSK-Adjuvanted-RSVPreF3-adults-etr.html) and Pfizer RSV vaccines (https://www.cdc.gov/vaccines/acip/recs/grade/Pfizer-Bivalent-RSVpreF-adults-etr.html).

¶ RSV-associated LRTD (RSVPreF3 trial): two or more lower respiratory symptoms (new or increased sputum, cough, and dyspnea) or signs (new or increased wheezing, crackles or rhonchi detected during chest auscultation, respiratory rate ≥20 respirations per minute, low or decreased oxygen saturation, and need for oxygen supplementation) for ≥24 hours (including one or more lower respiratory signs) or three or more lower respiratory symptoms for ≥24 hours.

** Manufacturer-calculated efficacy. Includes events >14 days after injection and person-time available from the manufacturer’s pivotal phase 3 trial. Estimates are adjusted for participant age and region.

†† Medically attended RSV-associated LRTD (RSVPreF3 trial): LRTD plus attendance at one or more inpatient or outpatient health care service. Estimates not included in per-protocol assessments.

§§ Persons with severe RSV illness requiring respiratory support (RSVPreF3 trial): RSV-associated illness requiring oxygen supplementation, positive airway pressure, or other types of mechanical ventilation. If participant was already receiving any of these, significant change or adaptation was considered.

¶¶ The limited number of hospitalizations, severe RSV illnesses, and deaths observed in the trial might have been partially due to limited enrollment of persons at highest risk for RSV disease including those who were frail, of advanced age, and those living in long-term care facilities and the exclusion of persons with immune compromise. The 2021–22 RSV season was also disrupted by the COVID-19 pandemic, and RSV incidence was lower than expected based on prepandemic surveillance studies.

*** Serious adverse events were defined as any untoward medical occurrence that resulted in death, was life threatening, required inpatient hospitalization or prolongation of existing hospitalization, resulted in persistent disability or incapacity, or was a congenital anomaly or birth defect.

††† RSV-associated LRTD (RSVpreF trial): the trial had two co-primary endpoints, defined as RSV lower respiratory tract illness (LRTI) with two or more lower respiratory signs or three or more lower respiratory symptoms (including new or worsened cough, sputum production, wheezing, shortness of breath, and tachypnea) lasting >1 day. For RSVpreF estimates in this report, LRTD refers to the RSVpreF trial endpoint of LRTI with three or more signs or symptoms.

§§§ Manufacturer-calculated efficacy. Includes events occurring >14 days after injection and person-time available from the manufacturer’s pivotal phase 3 trial. Estimates are not adjusted.

¶¶¶ Medically attended RSV-associated LRTD (RSVpreF trial): LRTD prompting any health care visit. Estimates not included in per-protocol assessments.

**** Severe RSV illness requiring respiratory support (RSVpreF trial): RSV-associated acute respiratory illness with new or increased oxygen supplementation or mechanical ventilation.

†††† The limited number of hospitalizations, severe RSV illnesses, and deaths observed in the trial might have been partially due to limited enrollment of persons at highest risk for RSV disease including those who were frail, of advanced age, and those living in long-term care facilities and the exclusion of persons with immune compromise. The 2021–22 RSV season was also disrupted by the COVID-19 pandemic, and RSV incidence was lower than expected based on prepandemic surveillance studies.

§§§§ Votes: 1) Adults aged 60–64 years may receive a single dose of RSV vaccine, using shared clinical decision-making (13–0 vote in favor, one abstention), and 2) Adults aged ≥65 years may receive a single dose of RSV vaccine, using shared clinical decision-making (nine to five in favor). Several ACIP members who voted no for shared clinical decision-making in adults aged ≥65 years were in favor of a routine recommendation for all persons in this age group. https://www.cdc.gov/media/releases/2023/s0629-rsv.html

¶¶¶¶
https://www.cdc.gov/coronavirus/2019-ncov/need-extra-precautions/people-who-are-immunocompromised.html

***** When administering more than one vaccine at the same clinical visit, providers should separate injection sites by at least 1 inch if possible and consider administering vaccines that are associated with an enhanced local reaction in separate limbs.
